# Comparison of Endogenous Endophthalmitis in Patients with and Without COVID-19 Infection

**DOI:** 10.1155/2022/6284569

**Published:** 2022-11-15

**Authors:** Ehsan Namvar, Mehdi Moallem, Mehrdad Afarid, Alireza Bolkheir

**Affiliations:** Poostchi Ophthalmology Research Center, Department of Ophthalmology, School of Medicine, Shiraz University of Medical Sciences, Shiraz, Iran

## Abstract

**Introduction:**

Endogenous endophthalmitis (EE) is an ophthalmic emergency that can have severe sight-threatening complications. Prompt diagnosis and aggressive treatment are central to the successful management of EE. In recent years, a significant increase has occurred in the number of cases of EE. Some of these individuals had a COVID-19 infection. The current study compared EE in patients with and without COVID-19 infection.

**Methods:**

All cases of EE admitted to Khalili Hospital between April 2020 and September 2021 were included in this prospective case-control study. Patients were divided into 2 groups: (i) the case group (EE patients with confirmed COVID-19 infection; *n* = 7) and (ii) the control group (EE patients without a history or evidence of COVID-19 infection; *n* = 7). Age, sex, presenting and final visual acuity, systemic diseases and risk factors for EE, anterior segment and fundus findings, hospitalization due to COVID-19, intensive care unit (ICU) admission, systemic steroid therapy, results of the sepsis workup, causative microorganism, types of treatment (pars plana vitrectomy vs. intravitreal or antifungal antibiotics), and follow-up period were recorded.

**Results:**

Twenty-four eyes of the 14 patients were included in this study, of which 9 were female. The mean age was 49.57 years. Follow-ups ranged from 1 month to 20 months (mean, 8 months). There were no statistically significant differences in age (*P* = 0.653), mean follow-up (*P* = 0.943), gender (*P* = 0.313), and clinical presentation (*P* = 0.409) between the case and control groups. Seven patients (50%) had positive intraocular culture results. Two out of 7 patients had a history of COVID-19 infection. The most common causative microorganism was Candida (4 patients [28.57%]; 6 eyes [25%]). No statistically significant differences were observed between the 2 groups in the need for vitrectomy (*P* = 1.000). The visual outcome between the 2 groups was similar (*P* = 0.179).

**Conclusion:**

The COVID-19 infection does not seem to affect the severity, visual outcomes, improvement rate, or vitrectomy rate of EE. Early diagnosis and management, especially pars plana vitrectomy, can prevent serious complications and save many eyes.

## 1. Introduction

Endogenous endophthalmitis (EE), as an intraocular infection, is the resultant form of hematogenous spreading from distant foci. It may enforce devastating systemic and ocular complications, although they are uncommon [1]. According to several reports, EE accounts for 2%–8% of all endophthalmitis cases [2–6]. EE is far less prevalent than exogenous endophthalmitis; the former is more likely related to a potentially life-threatening and underlying systemic source or risk factor for infection, such as recent hospitalization, intravenous drug use, or an indwelling catheter. More commonly, it happens in those with 1 or more contributors to relative immunosuppression, including malignancy, early or advanced age, diabetes mellitus (DM), or using corticosteroids or noncorticosteroid immunosuppressive agents [7].

Globally, bacterial infections account for most cases of EE, with gram-positive bacteria (particularly *Streptococcus* and *Staphylococcus* species [spp.]) happening more frequently in most settings and gram-negative bacteria (particularly *Klebsiella* spp.) being more prevalent in Asia [4]. Endogenous fungal endophthalmitis is mainly caused by Candida spp., which is related to the use of intravenous drugs and indwelling catheters. It predominantly occurs in industrialized countries, mostly the USA [8, 9]. EE caused by *Aspergillus* spp. shows far less prevalent fungal causes. However, it is distinguished by its common relation with a debilitating disease and also a preference for localizing under the retina [10–12].

The successful management of EE is based on aggressive treatment and prompt diagnosis, even though the prognosis is poor for many patients.

In the last 1 year, a considerable increment was found in the number of EE cases in our hospital. More importantly, the COVID-19 infection was found in half of these individuals.

## 2. Materials and Methods

The present study is a prospective case-control study. All cases of EE without exogenous causes (such as trauma, surgery, or intravitreal injection) who were admitted to Khalili Hospital (a referral eye hospital in the south of Iran, Shiraz) between April 2020 and September 2021 were included in this study.

Informed written consent was obtained from all participants and their legal guardian (for illiterate study participants). In accordance with the Declaration of Helsinki, this study was approved by the Ethics Committee of Shiraz University of Medical Sciences (IR.SUMS.REC.1401.148).

Post-operative endophthalmitis, post-traumatic endophthalmitis, postinjection endophthalmitis, and noninfectious uveitis were excluded from the study. Patients who had EE without a history or evidence of COVID-19 infection were considered the control group, and those who had EE with confirmed COVID-19 infection (positive polymerase chain reaction [PCR] or hospitalization due to clinical manifestations, especially pulmonary involvement in chest computed tomography [CT]) were considered the case group.

These data were collected from patients and their old charts. Some information, such as systemic diseases, confirmed COVID-19 infection (positive PCR or hospitalization due to clinical manifestations, especially pulmonary involvement in chest CT), severity of COVID-19, intensive care unit (ICU) admission, and systemic steroid therapy, was collected from old charts of patients (from internal medicine wards). Patients were categorized as having confirmed EE based on microbiological results and presumed EE based on clinical manifestations and responses to antibiotic and antifungal treatment. Age, sex, presenting and final visual acuity, systemic diseases and risk factors for EE, anterior segment and fundus findings, severity of COVID-19, hospitalization due to COVID-19, ICU admission, systemic steroid therapy, interval between COVID-19 infection and EE, results of sepsis workup, severity of EE, causative microorganism of EE, types of treatment (pars plana vitrectomy vs. intravitreal or antifungal antibiotics), duration of hospitalization due to EE, degree of anatomical and functional improvement, macular optical coherence tomography (OCT), and follow-up period were recorded. Best-corrected visual acuity (BCVA) is determined through the Snellen chart.

Statistical analysis was performed using SPSS version 18 (SPSS Inc, Chicago, Ill, USA) to compare the 2 groups. We compared the case and control groups using Mann-Whitney, chi-square, and *t* tests. *P* values <0.05 were considered statistically significant.

## 3. Results

Among the 24 eyes of the 14 patients in this study, 9 were women with a mean age of 49.57 years within the range of 30–84 years. Follow-ups ranged from 1 month to 20 months (mean, 8 months). No statistically significant differences were observed in age (*P* = 0.653), mean follow-up (*P* = 0.943), and gender (*P* = 0.313) between the case and control groups. Ten patients had bilateral endogenous fungal endophthalmitis. Seven of them had a positive history of COVID-19.

No patient was identified through routine screening. A primary diagnosis of endophthalmitis was performed in all 24 eyes (100%). At primary assessment, 22 eyes had diffuse posterior and anterior inflammation. There were no statistically significant differences between patients with and without COVID-19 infection (*P* = 0.409) regarding the clinical presentation. Among 10 patients with bilateral endophthalmitis, 2 patients had diffuse inflammation in 1 eye and focal posterior inflammation in the fellow eye. One of them had a history of COVID-19.

At least 1 connected systemic medical condition was found in all patients (Table 1). Eight patients (57.15%) had 2 or more risk factors. All patients were hospitalized in the past 6 months. The most prevalent risk factor was DM (8 patients [57.15%]). Four patients (28.57%) received immunosuppressive therapy. Seven patients (50%) had a history of COVID-19.

Positive intraocular culture results were found in 7 patients (50%). Two out of 7 patients had a history of COVID-19. The most prevalent primary diagnostic procedure was vitreous paracentesis in 24 eyes, yielding positive culture results in 11 eyes (45.83%; 8 eyes in the non-COVID-19 group and 3 eyes in the COVID-19 group). An alternative primary diagnostic procedure was vitrectomy, which was included in 14 (58.33%) out of 24 eyes. Seven out of these 14 eyes were in the COVID-19 group. There were no statistically significant differences between the 2 groups in the need for vitrectomy (*P* = 1.000).

The first most common causative microorganism was Candida (4 patients), and the second one was *Aspergillus*. One patient had a positive bacterial intraocular culture result (S. epidermidis). The microbiology of intraocular culture results from EE patients is shown in Table 2.

Along with positive intraocular culture results, positive culture results were found for 5 patients (35.71%) from nonocular samples (4 patients in the non-COVID-19 group and 1 patient in the COVID-19 group). Of these, blood cultures revealed positive results in 1 patient in the non-COVID-19 group (7.14%), and urine cultures showed positive results in 4 patients (28.57%). Echocardiography of patients showed a left ventricular clot in 1 patient (non-COVID-19 group) and vegetation in another patient (COVID-19 group).

Initial treatment consisted of a combination of ocular and systemic treatment in all patients. Ocular treatment included intravitreal injection in all eyes and vitrectomy with lensectomy or without it in 14 eyes (58.33%). All patients were initially treated with intravenous treatment (voriconazole). During the treatment course, all eyes received intravitreal injections. The agent used was voriconazole (50–100 *μ*g/0.1 mL).

Fourteen out of 24 eyes (58.33%) experienced pars plana vitrectomy during the treatment course. All of them received an antifungal injection during the pars plana vitrectomy.

Visual acuity was accessible for all patients at their last follow-up examinations.  Table 3 shows the visual outcome of EE patients. No statistically significant differences were found between the 2 groups in the visual outcome (*P* = 0.179).

In the last follow-up examination, a macular OCT was taken. The mean central macular thickness (CMT) in the non-COVID-19 and COVID-19 groups was 359.91 ± 191.40 and 297.75 ± 121.63, respectively. There were no statistically significant differences between the 2 groups in CMT (*P* = 0.386). In addition, the thickness of the different retinal layers was evaluated by OCT, but no statistically significant differences were detected between the 2 groups (Table 4).

No patient needs evisceration or enucleation. No phthisis bulbi were reported.

## 4. Discussion

EE is an uncommon intraocular infection. Risk factors for EE consist of immunosuppressive therapy, chronic diseases (such as DM, malignancies, renal failure, and acquired immunodeficiency syndrome), major surgeries, indwelling catheters, intravenous drug abuse, and pregnancy [9].

White et al. [13] showed an incidence of 26.7% of invasive fungal disease (most commonly Aspergillus) in patients with COVID-19 who were admitted to the ICU. In addition, they found that the use of corticosteroids increased the likelihood of the fungal infection. Similarly, in our study, all COVID-19 positive EE cases had received systemic steroids. However, in our study, only 2 out of 14 patients (14.28%) showed Aspergillus as a causative organism.

EE as an important complication in the setting of COVID-19 infection was analyzed in our study. The case and control groups were homogenous according to age, gender, and follow-up. There were no differences in the visual outcome, clinical improvement, severity, and rate of vitrectomy between the case and control groups. Although COVID-19 increases the prevalence of intravenous line-related candidemia 5-fold [14] and may increase EE, it does not seem to affect the severity, structural and functional outcomes, improvement rate, or vitrectomy rate of EE.

All patients presented at least several weeks after the resolution of COVID-19. This implies that the viral infection had been improved and that persistent immune suppression or even other comorbidities had led to EE.

Although the outcome of EE depends on the virulence and load of the microbial organism, the high index of clinical suspicion, early diagnosis, and management can save many eyes. In our cases, we were able to save the eyes of all the patients with a good visual outcome in 41.66% of eyes.

A systematic review showed that vitrectomy and intravitreal treatment in EE improved vision to better than 20/200 2 times more and reduced evisceration or enucleation 3 times more than intravitreal treatment alone [15]. Bilgic et al. reported good outcomes of early intervention for EE in the setting of COVID-19 infection [16]. Similarly, in our study, 14 out of 24 eyes needed vitrectomy during the treatment course, and they had good functional and structural outcomes.

Oren et al. study represented that statistically significant differences in the macula, ganglion cell layer, and inner nuclear layer can be detected 14 to 30 days after the onset of COVID-19 symptoms [17]. But our study showed no statistically significant differences in CMT and thickness of the different retinal layers in macular OCT between the 2 groups. It may be due to the long interval time between macular OCT taking and COVID-19 symptoms. Therefore, there were no differences in functional and structural outcomes between the COVID-19 and non-COVID-19 EE patients.

One of the limitations of our study was the small numbers of cases. So, larger studies will be needed in the future.

## 5. Conclusion

The most common causative microorganism in EE is Candida. The COVID-19 infection does not seem to affect the severity, visual outcomes, improvement rate, or vitrectomy rate of EE.

## Figures and Tables

**Table 1:** Comparison of systemic risk factors of EE patients with and without COVID-19.

Risk factors	Number in case group	Number in control group
Diabetes mellitus	3	5
Systemic steroids	7	0
Cancer	1	1
Systemic surgery	2	2
Urinary tract infection	3	4
Cushing syndrome	0	1
Cardiac disease (CABG, endocarditis)	2	0
Massive skin burn	0	1
Rheumatologic disease (RA/dermatomyositis)	1	1
Gastrointestinal disease (pancreatitis)	0	1
ICU admission	2	0

CABG, coronary artery bypass graft surgery; RA, rheumatoid arthritis.

**Table 2:** Comparison of microbiology of EE patients with and without COVID-19.

Organisms	Case group		Control group	
	No. of patients	No. of eyes	No. of patients	No. of eyes
Candida	1	1	3	5
Aspergillus	1	2	1	2
*Staphylococcus* epidermidis	0	0	1	1

**Table 3:** Comparison of visual outcomes of EE patients with and without COVID-19.

Visual acuity	No. of patients in case group	No. of patients in control group
≥20/200	8	6
≥20/50	8	2

**Table 4:** Comparison of different retinal layers thickness of EE patients with and without COVID-19.

Retinal layers	COVID-19	Number of eyes	Mean	Standard deviation	*P* value
RNFL	No	12	26.00	34.96	0.653
	Yes	12	20.12	14.37	

GCL	No	12	20.00	8.33	0.693
	Yes	12	22.00	11.58	

IPL	No	12	25.60	11.12	0.808
	Yes	12	24.25	11.75	

INL	No	12	22.50	6.86	0.140
	Yes	12	31.37	14.08	

OPL	No	12	25.50	5.95	0.843
	Yes	12	26.25	8.64	

ONL	No	12	87.50	26.58	0.051
	Yes	12	64.50	19.56	

RPE	No	12	14.72	2.19	0.543
	Yes	12	15.62	3.58	

Inner retina	No	12	231.27	111.06	0.366
	Yes	12	192.50	70.60	

Outer retina	No	12	82.75	6.18	0.725
	Yes	12	83.87	7.27	

Retina	No	12	359.91	191.40	0.386
	Yes	12	297.75	121.63	

RNFL; Retinal nerve fiber layer, GCL; Ganglion cell layer, IPL; Inner plexiform layer, INL; Inner nuclear layer, OPL; Outer plexiform layer, ONL; Outer nuclear layer, RPE; Retinal pigment epithelium.

## Data Availability

Relevant data will be available by the corresponding author upon reasonable request.
